# Social support among HIV-positive and HIV-negative adolescents in Umlazi, South Africa: changes in family and partner relationships during pregnancy and the postpartum period

**DOI:** 10.1186/s12884-015-0542-z

**Published:** 2015-05-17

**Authors:** Lauren M Hill, Suzanne Maman, Allison K Groves, Dhayendre Moodley

**Affiliations:** 1 Department of Health Behavior, Gillings School of Global Public Health, University of North Carolina at Chapel Hill, Chapel Hill, North Carolina USA; 2 Department of Sociology, American University, Washington, DC USA; 3 Department of Obstetrics and Gynaecology, Nelson R. Mandela School of Medicine, University of KwaZulu-Natal, Durban, South Africa

**Keywords:** Adolescent Health, Antenatal, Postpartum, HIV, South Africa, Qualitative Research

## Abstract

**Background:**

Pregnancy is common among adolescents in South Africa, yet the social experiences of adolescents during the pregnancy and postpartum period remain understudied in this context. We aimed to explore how adolescent women’s discovery and disclosure of both their pregnancy and HIV status affected their relationships with family members and sexual partners, with a particular focus on whether and how support changed throughout this time period.

**Methods:**

We conducted in-depth semi-structured interviews with 15 HIV-positive and HIV-negative adolescent women who were either pregnant or had delivered in the last 18 months from one urban clinic in Umlazi, South Africa. Interviews were audiotaped, transcribed, translated, and coded for analysis.

**Results:**

Young women described stress and instability in their relationships with family and partners during pregnancy and the postpartum period, though prior to and during HIV-status disclosure women generally experienced less stress than in disclosing their pregnancy to family members and partners. After a destabilizing period immediately following pregnancy disclosure, families became and remained the primary source of material and emotional support for the young women. Women discussed heightened closeness with their partners during pregnancy, but few women had close relationships with their partners postpartum. Support experiences did not differ by HIV status.

**Conclusion:**

Programs should be aware of the relative importance of pregnancy-related concerns over HIV-related concerns in this population of young women. Engaging family members is critical in ensuring social support for this population of young pregnant women, and in encouraging timely initiation of antenatal care.

## Background

Thirty-five percent of young women in South Africa under 21 years of age have been pregnant, and the majority (54%) of these pregnancies are unintended [1]. Fifty-seven percent of first births are self-reported as mistimed [2] among South African women of all ages, and an even greater proportion (79%) of first births are reported as mistimed among women under the age of 20 [3,4]. Unintended pregnancies, particularly among youth, are associated with a range of negative outcomes for mothers and their infants. Adolescent mothers in low-resource settings are at higher risk for adverse maternal and child health outcomes such as preterm delivery, low birth weight, neonatal mortality, and late spontaneous or induced abortions than their adult counterparts [5-10], and are disproportionately affected by poor mental health outcomes [11]. Depression, stress, and other negative mental health outcomes are problems in and of themselves, and also tend to be associated with worse clinical outcomes for women [12].

Despite the high prevalence of pregnancy among adolescents in South Africa, studies have documented widespread stigmatization of teenage pregnancy. Pregnancy during adolescence is often portrayed as a social problem, both a product and a cause of social ills such as school dropouts, inadequate parenting, and negative obstetric outcomes [13,14]. The stigmatization of adolescent pregnancy may cause weakening of social support [15], which during pregnancy and postpartum is integral to the health of both the adolescent mother and her child. Lack of social support has been linked to poor mental and physical health outcomes among young pregnant women [16-24]. In turn, negative mental health among mothers is linked to poor outcomes among their offspring, including preterm birth and low birth weight [25,26]. Social support confers further benefits for HIV-infected pregnant and postpartum adolescents, promoting adherence to antiretroviral therapy (ART) regimens which promote the health of the mother and protect against mother-to-child transmission of HIV [27,28].

Adherence in this population is of particular concern with WHO PMTCT guidelines version Option B+ becoming the standard of care in settings like South Africa, as low adherence in large populations could lead to increasing drug resistance and poor treatment outcomes [29]. The South African government issued new prevention of mother-to-child transmission (PMTCT) guidelines coming into effect in 2015 [30] in line with Option B+ recommendations from the WHO by which pregnant HIV-positive women initiate ART for life upon diagnosis regardless of CD4 count [31]. Many other countries are moving toward implementing these latest WHO guidelines in place of Option B, under which HIV-positive mothers continue antiretroviral therapy (ART) after breastfeeding cessation only if they have a CD4 count under 350 cells/μl [31].

With these new recommendations there will be more women in better health beginning ART than previously, highlighting to need to promote social support for HIV-positive young women as they initiate lifelong treatment that requires very high adherence. Support may help them to maintain sufficiently high adherence to maintain their own health and the health of their infant [27,28]. For HIV-negative young women, social support will continue to play an important role through pregnancy and the postpartum period to enhance the clinical outcomes for themselves and their babies, and to prevent future HIV infection in this high risk population [32]. Given the importance of social support in this population and the primary role that both partners and families play in providing support to young women, we need a better understanding of how partners and families provide or fail to provide support over the course of pregnancy and the postpartum period.

Much of the existing literature on social support during pregnancy focuses on the effect of partner support on young mothers’ health. Young women’s partners may be important sources of social support during pregnancy, as they are generally more accepting of their pregnancies than parents and other family members [33,34]. Strong partner support during pregnancy has been associated with better psychosocial outcomes for young women [11], but we have little understanding of the role that partner support plays during the postpartum period.

In addition, little is understood about how families support South African adolescents during both pregnancy and the postpartum period. The potential role of family in the South African context warrants attention given that 93% of South African adolescent mothers are unmarried and live with their family during pregnancy and the postpartum period [1]. In other contexts family social support among adolescent mothers has been seen to promote breastfeeding [35] and to be protective against postpartum depression [36].

We explored the social support received by pregnant and postpartum adolescents in one antenatal clinic in Umlazi Township, Durban, South Africa. We describe the relationships that young women have with family and partners during pregnancy and the postpartum period, how these relationships differ for HIV-positive and HIV-negative women, and discuss the implications of these social support patterns for the care of young women during this critical period.

## Methods

### Background and Setting

During the course of a behavioral intervention trial to enhance support for HIV positive and HIV negative pregnant and postpartum adult women [37] our study team recognized that we understood little about the clinical and psychosocial experiences of the youngest women in the clinic. In response to this observation, we conducted formative research with adolescent pregnant women in the clinic to document their experiences during and after pregnancy.

The study took place in Umlazi Township, Durban, which is the second largest township in South Africa [38] with an estimated population ranging from 300,000 [39] to nearly 2,000,000 [38]. This wide range is due to the fact that some estimates include informal settlers while others do not. In the township pregnant women receive antenatal care at one of 17 primary health care clinics in the township, are referred to the hospital for delivery, and return to the clinics for postpartum care. Women in the study were recruited from one primary health care clinic in the township. Ethical clearance for the study was obtained from the institutional review boards at the University of North Carolina at Chapel Hill and the University of KwaZulu-Natal. Written informed consent was obtained from all study participants.

### Participants

Fifteen participants aged 14-17 were purposively selected among clients from the clinic to capture experiences during both pregnancy and the postpartum period as well as HIV-positive and HIV-negative women’s experiences. Nine of these women were receiving antenatal services at the clinic, four of whom were HIV-positive and five were HIV-negative. The remaining six participants were receiving postpartum care at the clinic, two of whom were HIV-positive and four were HIV-negative. We purposively selected participants to represent both HIV-positive and HIV-negative women as we wanted to understand the extent to which support experiences varied by HIV status. We purposively selected women to capture both pre- and postnatal experiences to capture the long-term experience of women in ante- and postnatal care as we did not have the opportunity to conduct follow-up interviews. Women were recruited to participate in interviews until we reached data saturation in our key domains of interest.

The average age of both pregnant and postpartum participants was 16 years old (with ranges of 14-17 and 16-17, respectively). All women reported being in a relationship and their partners’ ages ranged from 18 to 26. The average participant had completed 10th grade (range grades 9-12), and half of the young women were currently attending school. The average gestational age of pregnant participants was 27 weeks (range 10-38 weeks), and the average age of infants born to participants in postpartum care was 3 months (range 1-4 months). All participants lived at home with their parents or guardians.

### Data collection

All interviews were conducted by two female local interviewers who were fluent in Zulu and English. Both interviewers were university graduates between 20 and 30 years old,and were trained to use a semi-structured guide outlining key interview questions. Interviewer training addressed qualitative research basics, interview skills including, building rapport, posing open-ended questions, probing, and active listening, and comprehension of the study research questions and interview guide.

The interview guide was developed by the study team to capture the clinical and social experiences of these adolescents as it related to both their pregnancy and their HIV status. Interview questions focused on pregnancy and HIV status discovery and disclosure experiences, participants’ relationships with family, partners, and friends during pregnancy and postpartum, and changes in these relationships related to their pregnancy and disclosure of HIV status.

### Data translation and analysis

To ensure accuracy of translation, all interview recordings were initially transcribed verbatim in Zulu by the interviewer who conducted the interview. An independent translator then translated the interview transcripts from Zulu to English. After translation, the English transcripts were reread for accuracy by the interviewers to ensure their fidelity to the original interviews.

We analyzed the data using qualitative descriptive approach [40]. The translated data were coded using Atlas.ti version 7. The first author was responsible for coding the interview transcripts using topical codes to categorize women’s relationships with their families and partners during pregnancy and the postpartum period. The first author shared code summaries and quotes with the author team, and discussions among all authors informed the interpretation of the data. In cases where initial author interpretations conflicted, consensus interpretations are presented here. After coding, we constructed matrices to assess whether and how support differed depending on whether women were HIV-infected or not, and whether they were currently pregnant or in the postpartum period. Finally, we constructed matrices to compare how support differed by relationship type (i.e. partner vs. family). Patterns and themes emerging from this process provide the foundation for the presentation of findings.

## Results

Young women described stress and instability in their relationships with family and partners during pregnancy and the postpartum period, though they generally described feeling less stress about disclosing their HIV status than disclosing their pregnancy to family members and partners. In disclosing their pregnancies, women faced many negative reactions from family members and more positive reactions from partners. After a destabilizing period immediately following pregnancy disclosure, families became and remained the primary source of material and emotional support for the young mothers. Women discussed heightened closeness with their partners during pregnancy, but few women had close relationships with their partners postpartum.

### HIV status disclosure

All women who tested HIV-positive discovered their status during their first antenatal visit which preceded their interview with study staff. All four pregnant HIV-positive women had disclosed their status to at least one family member or partner, though one had not disclosed her status to her partner and one had yet to disclose to her family. Despite initial concerns about disclosing their HIV status to family members and partners, most women received supportive reactions, as evidenced by this woman’s experience: “*I tested and found that I was positive and I was feeling really sad. I got home then I was able to tell my mother and sister and they were understanding, then I got used to it too,”* (HIV-positive postpartum). Of the six HIV-positive participants both pre- and postpartum, five were aware of the status of their partners who were all HIV-positive as well. Four women discovered their partner’s status upon disclosing their own status, and all who disclosed received supportive or neutral reactions from partners regarding their diagnosis.

Further into pregnancy and postpartum, women noticed no changes in relationships with their partners as a result of the disclosure of their HIV status: *“[My boyfriend] doesn’t discriminate against me and he loves me the way he used to love me… I don’t have a problem not to say I don’t care, I do care,”* (HIV-positive pre-partum). In general, women’s descriptions of support and relationship changes with family members and partners were much more colored by their pregnancies than by their HIV-status. Further, women’s social experiences related to their pregnancy were very similar for HIV-positive and HIV-negative women. For these reasons we have not disaggregated the findings presented below by HIV status.

### Pregnancy disclosure

All of the women interviewed reported that their pregnancies were unplanned, and they described being both surprised and disappointed when they learned they were pregnant: *“I very disappointed because I wasn’t expecting to fall pregnant. I wasn’t ready to fall pregnant, my pregnancy was a mistake,”* (HIV-negative postpartum). Women worried much more about disclosing their pregnancy to their parents and guardians and feared the negative reactions from family that they anticipated following disclosure. These fears were sometimes grounded in conversations that young women had had with parents and guardians in which they had been warned against pregnancy, as described by this young woman:

*“I’m afraid to tell my mother because things she says when we are sitting in the house, she says, ‘If you ever fall pregnant I will hit you and make sure that you abort the baby and get rid of you’.” – HIV-negative pre-partum*

The fear of negative reactions led young women to delay disclosure of the pregnancy. Many concealed the physical signs of pregnancy for as long as possible from their families. Women’s hesitation to disclose to family members proved to be justified in many cases, as in the majority of cases they responded to the news of the pregnancy with anger and in two cases temporarily ejected young women from the home. This young woman who was interviewed postpartum describes the angry reaction from her mother and acknowledged the disclosure moment as a pivotal time when her life changed forever:

“*Eventually my aunt noticed I was pregnant and told my mother. My mother asked me when the last time I had a period was. I said to her it’s been a while and she asked how long. I said it’s been 5 months. She asked ‘are you pregnant?’ I said yes. She started shouting and shouting and that was the beginning, that’s how life changed.” – HIV-negative postpartum*

In this example, and many others, it was the physical signs of pregnancy or changes in the menstrual cycle which prompted questions from family members and ultimately led to disclosure of the pregnancy. In a third of cases, young women described disclosing first to a sister who then helped her to share the news of pregnancy with parents. Sisters and other female relatives were able to shield women from the negative reactions of parents, by mediating the pregnancy disclosure, either by disclosing to the parents in the absence of the young woman or providing support to the young woman while she disclosed to her parents, as this young woman shares: “*There is a cousin, I thought would ask her to phone [my family] before I arrive [back in my village]… Yes, I want them to find out before I arrive,”* (HIV-negative pre-partum). The delay in disclosing the pregnancy to family had implications for women’s health care, as none of the women initiated antenatal care without having disclosed the pregnancy to at least one family member.

In contrast to disclosure experiences with family, none of the women expressed worry over disclosing their pregnancy to their partners. All women disclosed their pregnancies to their partners and all but one of the women’s partners reacted positively to the news :“*I just told him that I’m pregnant… he didn’t have a problem… he was happy, but he was surprised,”* (HIV-positive pre-partum). In several cases, young women found the excitement over the pregnancy from partners to be the complete opposite of their own feelings: “*He was excited…he has never had a child anyway so he was happy…. but I wasn’t happy,”* (HIV-positive pre-partum).In some cases women attributed their partner’s positive reaction to the news of pregnancy to the fact that they were older and thus more prepared to become parents than the women themselves.

### Relationships during pregnancy

Following the immediate stress associated with disclosure of the pregnancy, most women’s relationships with family stabilized. All women continued to live at home and most felt safe and supported in the home with regard to their pregnancy. Sisters or sister-like figures (i.e. female relatives of similar age) continued to be an important source of support for many participants as this woman describes:

“*I am very close to my sister; she is the one I speak to about important things. I sat down with her and told her that something has happened I don’t know what to do… I was able to tell my sister about the problem and ask her what should I do?”* - HIV-positive postpartum

Like this young woman, most participants were able to confide in and seek guidance from a sister figure. Few described similar levels of emotional closeness to schoolmates or other female friends. Sisters often provided material support as well, particularly when parents were unable or unwilling to buy young women what they needed during this period: *“My sister helps me with other things when my mother can’t… what I can’t get from my mother I am able to get from my sister,”* (HIV-negative postpartum).

Despite the restored closeness with family that many expressed later in pregnancy, many women felt they had disappointed their family by becoming pregnant at a young age. Most participants explained that their family had invested both money and hope in the prospect of their graduation from secondary school, and felt that they had disappointed their families’ hopes for their education by becoming pregnant. All of the women left school either during pregnancy or upon the birth of their child and many described stress associated with the withdrawal. These experiences triggered fears about being able to complete their education. Women themselves felt that the pregnancy was a major setback, if not an end to the hope of achieving this goal, and they described the hopes of their families also being dashed by the pregnancy.

“*I felt that I had failed my mother as a parent. For sure when a parent has children she wishes something for her children but since I have fallen pregnant even if I still do succeed in life it won’t be as my parents had wished it would have been like for me if I hadn’t had a baby.”* – HIV-negative postpartum

Despite the anxiety that some women faced in family relationships, during pregnancy many women enjoyed close, supportive relationships with their partners. In many cases women explained that the pregnancy brought them closer to their partner, reflected by more frequent contact with their partner as described by this woman:

“*We have a good relationship now… a month used to end without me seeing him, now a week doesn’t even end. Even when I don’t ask him to come by my house he still comes by to see me.”* – HIV-positive pre-partum

Families supported and encouraged increased closeness between women and their partners during pregnancy. For about half of the women the pregnancy led to plans of marriage with their partners, though none had actually married their partner by the time of these interviews.

During pregnancy many partners began to provide material support to the young women in ways that they had not done before. Partners began to take on a provider role, supporting women financially in the way that their parents had done before the pregnancy, as this woman explains: “*He is also important in helping me. If my mother were to die he can be important in acting like my parent, by clothing me and supporting my baby,”* (HIV-negative pre-partum). While not all women had partners who were financially capable of providing material support, all described being emotionally supported by their partner during pregnancy: “*My boyfriend is important because he has made me pregnant because I cannot face this thing alone so he is always with me all the time supporting me,”* (HIV-negative pre-partum).

However, not all women were satisfied with the material support received from their partners*.* Women and their families expected the father of the child and his family to provide symbolic gifts of acknowledgment of paternity, often in the form of livestock. In the cases where partners and their families failed to fulfil these expectations, women were disappointed and ashamed of being a financial burden on their families. Such failures to provide financial support were also related to partners and their partners’ families denying paternity of the child, as this woman explains:

*“We then went to the baby’s father’s home and the father denied that this baby is his. After delivery we went there again and his family said it’s their baby, but my heart is still broken because of that thing.”* – HIV-negative postpartum

### Postpartum relationships

During the postpartum period, family support continued to play an important role for young women, and partners tended to be less supportive than they were during pregnancy. For most women, parents and other family members were the primary source of material support for the new mother and her child.

*“If I need anything concerning the baby [my father] helps me, and my mother as well… Whenever I have something hurting me I’m able to talk about it with my mother, maybe when my boyfriend is hurting me, like when he didn’t want to be responsible for the baby I was able to talk to my mother… My mother and father are the ones who support my baby.* “– HIV-negative postpartum

Even when the partner continued to play a role in the young woman’s life, the woman’s family was still the primary source of financial support and a major source of emotional support. Further, some families who had been less supportive during pregnancy became more supportive after delivery.

“*My mother supports me in a lot of different ways… it was different when I was pregnant, it was like I was not even her child, and I was not in her heart. Now she is doing everything because even when I want to go and immunize the baby she is the one that gives me bus fare.”* – HIV-positive postpartum

Disappointment in the level of partner support was more commonly expressed in the postpartum period than during pregnancy. While all of the women lived with and were financially supported by a parent or guardian, many expressed that they had expected to depend fully on their partners for emotional and financial support after the birth of their child. When these expectations were not met, women expressed disappointment toward their partner and shame at placing a financial burden on their family.

“*It really upsets me to know that the father of my baby will impregnate me then desert me and the baby… [My parents] were upset, because they thought that they had sent me to school then the next thing they know I come back with a baby and that baby is not being supported by the father. Instead it has become their burden.”* – HIV-negative postpartum

Other young women who were not supported by their partners felt this double burden of disappointment in their partners and the guilt of placing the financial burden of childrearing on their parents. In addition to the lack of material support in light of the new burden of supporting a new-born, there was a noticeably lower level of closeness and emotional support received from partners.

“*I would love for him to acknowledge that I’m the mother of his child and acknowledge that he is the father of my child that’s all, not to say that we are dating or not. Nothing bad has happened it’s just that what I felt for him is finished.”* – HIV-negative postpartum

Though not all experiences with partners postpartum were negative, nearly all women described the importance of their family over their partner: “*He is important but not more than my family because obviously family gets first preference,”* (HIV-positive postpartum) In some cases this may have been due to a growing distance between the woman and her partner.

“*There have been changes [in our relationship] since late in my pregnancy. Before I fell pregnant we got along just fine… but in the end things got bad, we are like different people… now that I have given birth we get along like friends.”* – HIV-negative postpartum

Two women discussed ongoing close, supportive relationships with their partners with continued intimacy: “*He is able to support the baby… if I have a problem I tell him and he is able to help me solve it*,”(HIV-positive postpartum). Although a number of participants had discussed the prospect of marriage with their partners whilst pregnant, this topic of conversation was rarely discussed in the postpartum period.

## Discussion

Our findings underscore the important role that families play in providing social support to HIV-positive and HIV-negative adolescent women during pregnancy and the postpartum period in Umlazi, South Africa. Though the young women we interviewed experienced some instability with family immediately around pregnancy disclosure, families were women’s primary source of support in the long-term. Sisters, or sister-like figures, were particularly important in providing emotional support, and in some cases, additional material support for young women. Though some partners denied paternity, a distressing phenomenon for young mothers observed elsewhere in the South African context [41], most of the women we interviewed described their partners as supportive during pregnancy and less so during the postpartum period. This decline in relationship satisfaction postpartum reflects trends seen in other contexts, and studies indicate that such declines might be even more dramatic in couples with unplanned pregnancies as in this case [42,43].

Our findings point to the relative social impact of pregnancy over HIV status in young women’s immediate experiences. Pregnancy disclosure was consistently more stressful and problematic for young women than HIV disclosure. Further, HIV-positive young women generally received high levels of support with regard to their HIV status from family members and partners. Similar experiences have been observed in other South African studies in the adult population [44-47]. This comparatively supportive experience among HIV-positive women may be related to a perceived normalization of HIV/AIDS in the South African context. In communities such as the Durban metropolitan area, where the antenatal prevalence of HIV was 39% in 2012 [48], being HIV positive is coming to be viewed as normal [44]. Further, the success of ART has helped to shift perceptions of HIV in South Africa away from a death sentence toward a manageable chronic condition [46,47], contributing to the decline seen in stigmatization of people living with HIV/AIDS in South Africa [45,47]. The dominance of pregnancy-related concerns over HIV-related concerns in this population has been echoed in another study from KwaZulu-Natal [49]. As pregnancy has more immediate implications for young women’s lifestyles and higher visibility, it is likely that pregnancy will continue to have greater immediate social implications in this population than HIV. For these reasons efforts to promote HIV-related care among these women need to take into account the competing concerns related to unintended pregnancy and disclosure of pregnancy that young mothers may be facing.

Our findings have additional implications for the clinical care of adolescents. Anticipation of family reactions to pregnancy disclosure and subsequent negative reactions caused significant stress, and in some cases led young women to avoid disclosing the pregnancy for as long as possible. Women often waited for family members to notice the physical signs of pregnancy and often relied on family members to help them initiate care. As such, the delay in disclosing their pregnancy status also led to a delay in initiating antenatal care.

Given the central role of the family in triggering women’s initiation of antenatal care, it is critical to develop programs that encourage open dialogue between adolescent women and parents or guardians earlier in pregnancy. Such programs could be modeled after interventions encouraging parent-teen communication to reduce sexual risk behaviors which have shown success the domestic setting [50]. As seen in these women’s narratives, sisters or sister-like figures played a key supportive role during pregnancy and the postpartum period. Programs could leverage the role of sisters in assisting with pregnancy disclosure when appropriate and find ways to help young women reach out to them earlier. Future studies should seek to understand how to moderate negative reactions on the part of parents toward their daughters’ pregnancies and thus reduce women’s fear of disclosure.

There have been many efforts to date in South Africa to understand how to engage partners in PMTCT [51-56], but few efforts to involve parents and other family members in young women’s care [57]. Further, there have been few interventions addressing the needs of adolescent women in antenatal and postnatal care. Future interventions and research with this population should recognize family as a primary source of social support both before and after childbirth, and should seek to understand how to better engage families of adolescent women during this time. Improving social support received by young mothers could promote their physical and mental health [16-24], increase the likelihood of positive birth outcomes [25,26], and encourage their adherence to ART [27,28].

This study is not without limitations. With a purposively selected sample, our findings cannot be generalized to a wider population. As women were sampled from an urban clinic in one municipality, we cannot say to what extent these findings represent experiences of young women in other regions of the country, particularly rural regions. The purpose of these interviews was not to produce generalized findings, but rather to describe in depth the experiences of adolescent mothers in this setting. Young women’s family members and partners were not interviewed, thus we cannot speak to their perspectives. Future research should seek to understand the adolescent pregnancy and parenthood experience from family and partner perspectives, family and partner needs during this period, and the role they can play in promoting the health of both mother and child. The fact that only one person, the first author, coded the data could have limited triangulation of coding decisions. As codes used were topical rather than interpretive, there was less room for subjectivity than there would have been had the codes been more interpretive. Further, given that all women were recruited from one clinic, it is difficult to assess the extent to which our findings are transferable to other populations and settings. The women interviewed come from a large and important population of pregnant and parenting adolescents in urban and peri-urban greater Durban. Also, our findings related to family and partner support are echoed in other studies from South Africa, suggesting that our findings are transferable to other populations of adolescent mothers in South African Townships [1,2]. Lastly, the transcripts were translated from Zulu to English and as with any translation there was undoubtedly some level of information and understanding that was lost during the translation process. We included measures of quality control to check the quality of translation and minimize information loss.

These interviews provide insight into the complex and varied experiences of HIV-positive and HIV-negative young pregnant and parenting women in urban KwaZulu-Natal. Even among these varied experiences common themes emerged, most notably the importance of social support from family members through pregnancy and the postpartum period and the relative stability of this support in comparison to that received from partners. These findings point to intervention implications to improve clinical outcomes for these women. Ensuring sufficient, stable social support has important implications for mental and physical health outcomes among these women and their offspring [16-18,20-26], and for ART treatment outcomes [29]. Engaging parents, sisters, and other family members is critical in ensuring social support and in encouraging timely initiation of antenatal care for this vulnerable population. Our results further point to the relative importance of pregnancy-related concerns over HIV-related concerns in the minds of these young women during pregnancy and the postpartum period.

## Conclusion

Programs aiming to improve perinatal care in this population should be aware of the relative importance of pregnancy-related concerns over HIV-related concerns in this population of young women. Engaging family members is critical in ensuring social support for this population of young pregnant women, and in encouraging timely initiation of antenatal care.

## Abbreviations

HIV: Human Immunodeficiency virus
PMTCT: Prevention of mother to child transmission
ART: Antiretroviral therapy
